# Supplementary material for: Does Enhanced Structural Maturity of hiPSC-Cardiomyocytes Better for the Detection of Drug-Induced Cardiotoxicity?
Source: Biomolecules. 2023 Apr 14;13(4):676. doi: 10.3390/biom13040676 (PMC10135569; doi:10.3390/biom13040676)
Supplement: Supplementary file 1 [file biomolecules-13-00676-s001.zip › Biomolecules_2134105_Suppl Materials for sub.pdf]

Does enhanced structural maturity of hiPSC-cardiomyocytes better for the detection of drug-induced cardiotoxicity? (Van de Sande, Ghasemi et al.)

Supplementary Tables

Supplementary Table S1: List of the 11 reference compounds used in the study.

Compound	*Target*	*Function*	*CAS-#*	*Free Cmax (µM)*	*concentrations (µM)*
Quinidine	hERG and Nav	Inhibitor	56-54-2	0.92	0.3, 1, 3, 10
PA-6	Kir2.1	Inhibitor	500715-03-7	N/A	0.03, 0.1, 0.3, 1
Levosimendan	Troponin C	Activator	141505-33-1	0.1	0.1, 0.3, 1, 3
Milrinone	Phosphodiesterase-3	Inhibitor	78415-72-2	2.32	0.3, 1, 3, 10
Carbachol	M2 receptor	Agonist	51-83-2	N/A	0.03, 0.1, 0.3, 1
Oxotremorine	M2 receptor	Agonist	63939-65-1	N/A	0.1, 0.3, 1, 3
Dobutamine	β1-adrenergic receptor	Agonist	34368-04-2	0.08	1, 3, 10, 100
Isoprenaline	Beta1 adrenergic receptor	Agonist	7683-59-2	N/A	0.01, 0.03, 0.1, 0.3
Digoxin	Na^+/K^+ ATPase	Inhibitor	20830-75-5	0.002	0.1, 0.3, 1, 3
Diltiazem	Cav	Inhibitor	42399-41-7	1.45	0.01, 0.03, 0.1, 0.3
Dofetilide	hERG	Inhibitor	115256-11-6	0.0027	0.01 (positive control)
Nimodipine	Cav	Inhibitor	66085-59-4	0.069	0.1 (positive control)
DMSO 0.1%	N/A	Vehicle	67-68-5	N/A	0.1% (vehicle control)

Supplementary Table S2: Overview of the relevant parameters of the two models at baseline (DMSO 0%) and after DMSO 0.1% treatment as vehicle control.
Data are expressed as mean±SEM.

Model	*Control*	*Concentration*	*CL (ms)*	*Trise (ms)*	*APD_{30} (ms)*	*APD_{90} (ms)*	*CtAmp (AU)*	*CtD_{50} (ms)*
FM	DMSO	0%	1472±26.7	7±0.3	209±5	488±9.6	704±15.3	335±7.8
	DMSO	0.10%	1340±26.3	7±0.3	201±4.4	467±8.8	816±14.2	356±5.9
MM	DMSO	0%	1368±26	6±0.3	236±3.6	462±10.3	307±14.6	349±4.4
	DMSO	0.10%	1301±24.2	6±0.3	237±4.2	456±9.4	345±18.6	379±5.1

FM: fibronectin matrix. MM: maturity Matrix.

Supplementary Table S3: Overview of the effect of the 11 reference compounds on the relevant parameters of FM monolayers.

Compound	*Concentration (µM)*	*CL (%DD)*	*Trise (%DD)*	*APD_{30} (%DD)*	*APD_{90} (%DD)*	*CtAmp (%DD)*	*CtD_{50} (%DD)*
Quinidine	0.3	106±0.7*	103±14	105±2.9	115±2.5*	66±15.3	111±6.3
	1	114±2.4*	135±8.3*	104±1.4*	122±1.4*	162±50.3	Q
	3	Q	Q	Q	Q	Q	Q

	10	Q	Q	Q	Q	Q	Q
Diltiazem	0.01	102±1.1	105±17	98±1.9	96±1.1	142±27.5*	103±2.8
	0.03	103±1.3	103±12.5	92±1.5*	92±0.9*	127±27.9	95±1.4*
	0.1	96±0.8*	91±12	76±2.7*	78±2.2*	108±33.5	82±1.6*
	0.3	85±0.5*	116±13.8	60±1.3*	62±1.1*	59±15.4	68±1.4*
Milrinone	0.3	96±1.6	80±13.1	96±1.8	96±1.2*	103±10.9	101±1.7
	1	99±0.8	74±13.3	100±1	96±0.7*	116±13.2	104±2.4
	3	100±0.7	68±7.3*	96±1.9	94±0.4*	100±8.4	103±1.7
	10	103±0.6*	106±26.8	92±2*	95±1.1*	81±5.7	101±1.7
Levosimendan	0.1	95±1.4*	97±22.3	102±2.2	96±1.7	125±20.8	94±3.2
	0.3	93±1.1*	80±13.5	101±2.9	94±1.1*	94±9.6	100±2.4
	1	93±1.6*	65±9.5	94±3	93±0.6*	155±32.5	102±2.7
	3	97±1.2*	101±24.9	99±3.9	94±1*	122±26.6	101±1.8
Digoxin	0.1	98±0.7*	100±21.3	84±1.8*	95±0.6*	88±6.1	90±1.3*
	0.3	98±0.9	74±9.2*	65±2.8*	88±2.1*	18±11.1	51±15.9
	1	Q	Q	Q	Q	Q	Q
	3	Q	Q	Q	Q	Q	Q
Carbachol	0.03	99±2.6	104±11.8	97±2.3	99±1.4	94±15.9	104±2.1
	0.1	105±0.6*	125±18.5	104±2.6	100±1.2	85±10.4	103±2
	0.3	109±1*	118±17.5	102±2.9	103±1*	102±21.3	108±2.1*
	1	114±0.5*	95±17.7	103±1.6	110±1.1*	109±13.8	107±3.7
Cetirizine	0.1	95±2.2	154±32.6	96±2.7	94±2.7	72±13.3	101±2
	0.3	98±3	127±16.8	100±2.6	94±2.2	87±14.6	97±3
	1	95±0.9*	114±19.9	101±2.2	91±1.6*	129±32.9	102±1.6
	3	98±0.9	122±8.8	99±3.8	92±1.9*	112±20.4	98±2.3
Oxotremorine	0.1	117±1.3*	155±41.3	106±2.3*	103±1.3	140±19.7	108±1.9*
	0.3	122±1.8*	145±24.3	111±3.3*	105±2.8	105±16.5	112±2.5*
	1	121±2.9*	117±13.3	110±4.4*	111±1.7*	117±14.5	116±2.8*
	3	111±3.6*	132±11.2	107±3.4*	101±2.8*	166±17.6*	110±1.6*
Dobutamine	1	76±0.4*	86±5.3	84±1.4*	89±0.9*	93±10.7	79±1.5*
	3	77±0.9*	100±18.9	77±1.5*	86±0.8*	99±8.4	75±2*
	10	81±0.8*	131±21.5	90±2*	91±1.9*	82±3.2*	82±2.2*
	100	Q	Q	Q	Q	Q	Q
PA-6	0.01	101±0.8	101±17.4	108±3.6	104±1.1*	103±8.8	104±1.7
	0.1	98±2.3	70±6.1*	99±3	107±1.4*	111±14.2	105±1.8*
	0.3	101±2.5	89±10.8	105±2.4	111±0.8*	105±10.2	106±2.1*
	1	108±1.4*	128±15.8	110±1.3*	116±1.3*	111±8.1	112±2.5*
Isoprenaline	0.01	90±1.4*	80±7.3	97±2	94±1.9*	94±5.2	94±1*
	0.03	74±0.6*	97±8.6	85±1.7*	77±0.6*	90±8.3	88±4.6
	0.1	56±0.6*	106±4.5	68±0.8*	60±1*	125±17.9	76±2.8*
	0.3	49±0.6*	106±8.1	62±1*	52±0.5*	68±8.5	58±1.6*

*: $p<0.05$. Q: quiescent (beating stop). Data are expressed as mean±SEM, calculated as delta over DMSO 0.1%.

Supplementary Table S4: Overview of the effect of the 11 reference compounds on the relevant parameters of MM (maturity Matrix).

Compound	*Concentration (µM)*	*CL (%DD)*	*Trise (%DD)*	*APD_{30} (%DD)*	*APD_{90} (%DD)*	*CtAmp (%DD)*	*CtD_{50} (%DD)*
Quinidine	0.3	106±1.6*	111±15.7	104±1.4	108±1.2*	118±21.6	107±2.7
	1	114±1.5*	123±15	100±1.2	115±0.8*	99±13.1	97±2.5
	3	124±1.3*	188±23.3*	103±2.1	121±1.2*	106±11.4	105±1.5*
	10	Q	Q	Q	Q	Q	Q
Diltiazem	0.01	98±0.9	86±16.4	100±1.9	96±0.6*	101±11.1	105±2.7

	0.03	96±0.9*	79±4	91±1.2*	90±0.7*	94±7.8	99±2.6
	0.1	83±0.6*	79±14.3	80±1.6*	77±1.2*	93±6.2	81±1.2*
	0.3	71±0.5*	74±8.9	65±0.8*	62±0.9*	73±5.8*	61±1.9*
Milrinone	0.3	97±0.2*	112±18.8	96±1.6	97±1.1	131±17.5	100±1
	1	100±0.6	125±11.4	97±0.7*	99±0.5	115±6.3	105±1.8
	3	101±0.5*	132±27.3	97±1.4	102±1.4	76±9.1	104±1.5
	10	103±0.2*	102±7	95±1*	100±0.9	129±28.5	100±1.4
Levosimendan	0.1	99±0.8	107±17.8	97±1.4	96±1.2*	120±12.4	104±2
	0.3	97±0.7*	121±16.3	100±2.2	100±1	95±8.1	102±1.3
	1	97±0.7*	96±7.8	95±1.7*	98±0.9	199±27.2	104±2
	3	98±0.5*	114±10.2	93±1.9*	98±0.9	167±33.5	103±2.3
Digoxin	0.1	102±0.4*	127±10.8	91±1.2*	101±1.3	127±26.2	97±2
	0.3	100±0.8	161±38.2	68±2.5*	89±1*	12±3.3*	43±1.8*
	1	Q	Q	Q	Q	13±5.3	40±3.6
	3	Q	Q	Q	Q	7±1.9	43±3.4
Carbachol	0.03	106±0.3*	186±29	105±2.2	103±1.8	86±6.4	98±1.5
	0.1	115±0.6*	144±20.7	106±2.4	107±1.1*	135±12.9	102±1.8
	0.3	122±0.9*	143±22.1	111±1.9*	111±1.1*	118±12.9	111±2.1*
	1	131±0.7*	143±17.8	120±2.3*	118±1*	114±18.4	118±1.5*
Cetirizine	0.1	100±1	146±16.9	90±1.8*	93±0.9*	92±6.9	98±1.4
	0.3	105±0.9*	90±11.2	90±1.2*	93±1.1*	93±5.6	97±1.2
	1	101±1.2	97±10.3	91±1.4*	93±0.9*	118±14.2	98±1.2
	3	106±2.9	100±8.9	91±1.9*	93±0.6*	87±5.3	97±2.4
Oxotremorine	0.1	111±2.8*	124±18.7	104±1.7*	104±1*	106±11.7	116±2.1*
	0.3	115±3.3*	144±26.3	110±3.4*	108±1.1*	97±12.9	117±1.1*
	1	120±1.4*	117±17	110±2.8*	114±1.1*	105±10.2	118±1.6*
	3	123±1.1*	158±32	115±3.8*	113±1.2*	103±7.4	120±2.3*
Dobutamine	1	75±0.7*	91±7.1	89±1.2*	91±1*	101±11.6	82±1.1*
	3	76±0.5*	109±14.1	85±1.3*	91±0.9*	89±6.3	78±1.2*
	10	81±0.6*	146±13.9*	86±1.9*	96±0.9*	98±7.5	77±1.3*
	100	Q	Q	Q	Q	Q	Q
PA-6	0.01	97±0.7*	119±25.1	107±1.9*	103±1.5	127±13	101±1.3
	0.1	100±0.5	74±17.9	105±1*	111±2.2*	99±11.2	103±1.1*
	0.3	102±0.8*	96±12.7	107±1.9*	111±1*	135±22.2	105±1.9*
	1	108±2.4*	138±14.2	107±1.5*	111±1.7*	114±9.8	107±1.2*
Isoprenaline	0.01	90±0.9*	146±13.3*	92±0.8*	96±0.8*	123±14.4	96±2.3
	0.03	77±0.6*	115±12.9	83±0.9*	86±1*	112±11.7	88±2.3*
	0.1	63±0.8*	93±5.5	68±1.5*	68±1.2*	121±16.1	68±2.7*
	0.3	56±0.8*	129±10.9	62±0.8*	61±0.9*	105±14.9	57±1.8*

Data are expressed as mean±SEM, calculated as delta over DMSO 0.1%. *: $p<0.05$. Q: quiescent (beating stop).

Supplementary Table S5: Overview of the differences in the relevant parameters between FM and MM monolayers.

Compound	*Culture*	*Sample size*	*CL (%DD)*	*Trise (%DD)*	*APD_{30} (%DD)*	*APD_{90} (%DD)*	*CtAmp (%DD)*	*CtD_{50} (%DD)*
Quinidine (1µM)	*FM*	*8*	*114±2.4*	*135±8.3*	*104±1.4*	*122±1.4*	*162±50.3*	*106±1.8*
	MM	*8*	*114±1.5*	*123±15*	*100±1.2*	*115±0.8**	*99±13.1*	*97±2.5**
Diltiazem (0.1µM)	*FM*	*8*	*96±0.8*	*91±12*	*76±2.7*	*78±2.2*	*108±33.5*	*82±1.6*
	MM	*8*	*83±0.6**	*79±14.3*	*80±1.6*	*77±1.2*	*93±6.2*	*81±1.2*
Milrinone (10µM)	*FM*	*8*	*103±0.6*	*106±26.8*	*92±2*	*95±1.1*	*81±5.7*	*101±1.7*
	MM	*8*	*103±0.2*	*102±7*	*95±1*	*100±0.9**	*129±28.5*	*100±1.4*
Levosimenadan (3µM)	*FM*	*8*	*97±1.2*	*101±24.9*	*99±3.9*	*94±1*	*122±26.6*	*101±1.8*
	MM	*8*	*98±0.5*	*114±10.2*	*93±1.9*	*98±0.9**	*167±33.5*	*103±2.3*
Digoxin (0.1µM)	*FM*	*8*	*98±0.7*	*100±21.3*	*84±1.8*	*95±0.6*	*88±6.1*	*90±1.3*
	MM	*8*	*102±0.4**	*127±10.8*	*91±1.2**	*101±1.3**	*127±26.2*	*97±2**
Carbachol (1µM)	*FM*	*8*	*114±0.5*	*95±17.7*	*103±1.6*	*110±1.1*	*109±13.8*	*107±3.7*
	MM	*8*	*131±0.7**	*143±17.8*	*120±2.3**	*118±1**	*114±18.4*	*118±1.5**
Cetirizine (3µM)	*FM*	*8*	*98±0.9**	*122±8.8*	*99±3.8*	*92±1.9*	*112±20.4*	*98±2.3*
	MM	*8*	*106±2.9*	*100±8.9*	*91±1.9*	*93±0.6*	*87±5.3*	*97±2.4*
Oxotremorine (3µM)	*FM*	*8*	*111±3.6*	*132±11.2*	*107±3.4*	*101±2.8*	*166±17.6*	*110±1.6*
	MM	*8*	*123±1.1*	*158±32*	*115±3.8*	*113±1.2*	*103±7.4**	*120±2.3**
Dobutamine (10µM)	*FM*	*8*	*81±0.8*	*131±21.5*	*90±2*	*91±1.9*	*82±3.2*	*82±2.2*
	MM	*8*	*81±0.6*	*146±13.9*	*86±1.9*	*96±0.9*	*98±7.5*	*77±1.3**
PA-6 (1µM)	*FM*	*8*	*108±1.4*	*128±15.8*	*110±1.3*	*116±1.3*	*111±8.1*	*112±2.5*
	MM	*8*	*108±2.4*	*138±14.2*	*107±1.5*	*111±1.7*	*114±9.8*	*107±1.2*
Isoprenaline (0.1µM)	*FM*	*8*	*56±0.6*	*106±4.5*	*68±0.8*	*60±1*	*125±17.9*	*76±2.8*
	MM	*8*	*63±0.8**	*93±5.5*	*68±1.5*	*68±1.2**	*121±16.1*	*68±2.7*

Data are expressed as mean±SEM, calculated as delta over DMSO 0.1%. *: $p<0.05$.
