# Supplementary material for: Does Enhanced Structural Maturity of hiPSC-Cardiomyocytes Better for the Detection of Drug-Induced Cardiotoxicity?
Source: Biomolecules. 2023 Apr 14;13(4):676. doi: 10.3390/biom13040676 (PMC10135569; doi:10.3390/biom13040676)

Suppl. Figure S2:

(a) 1µM Quinidine

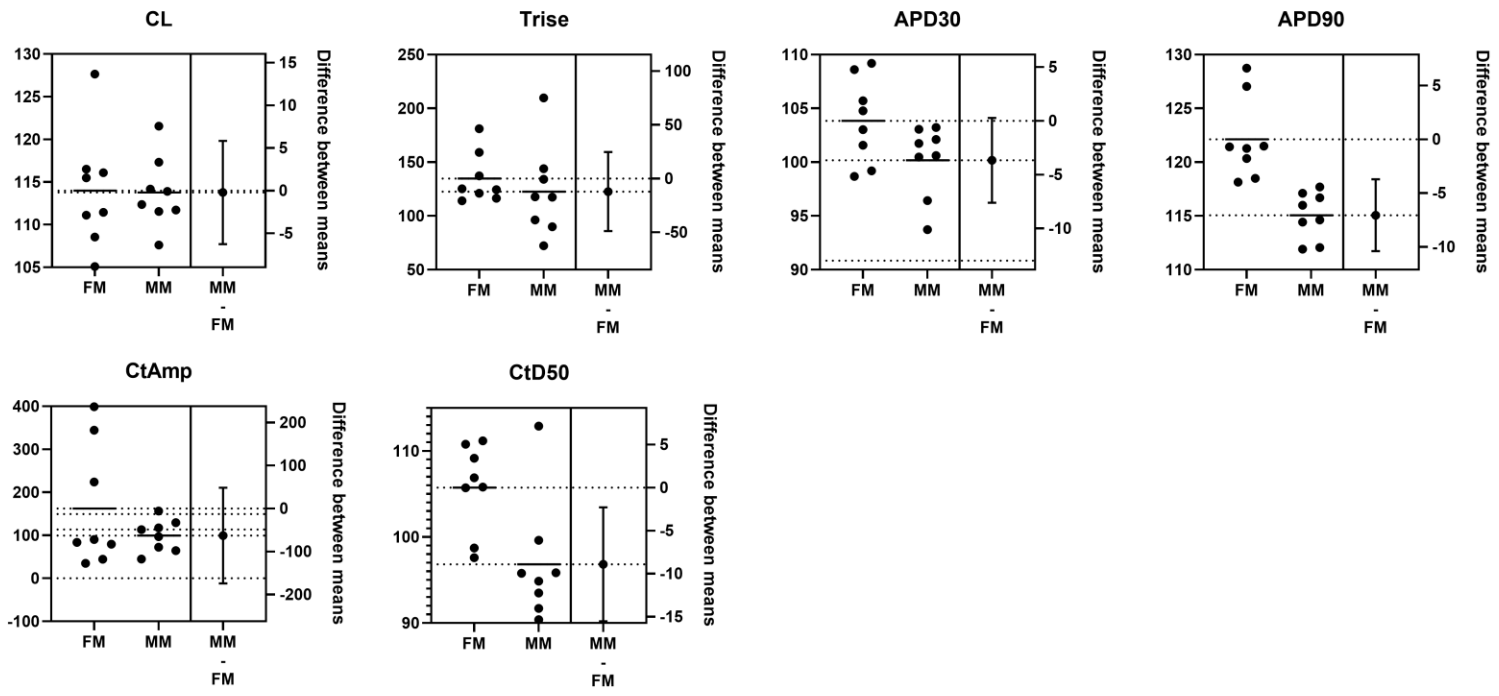

(b) 0.1µM Diltiazem

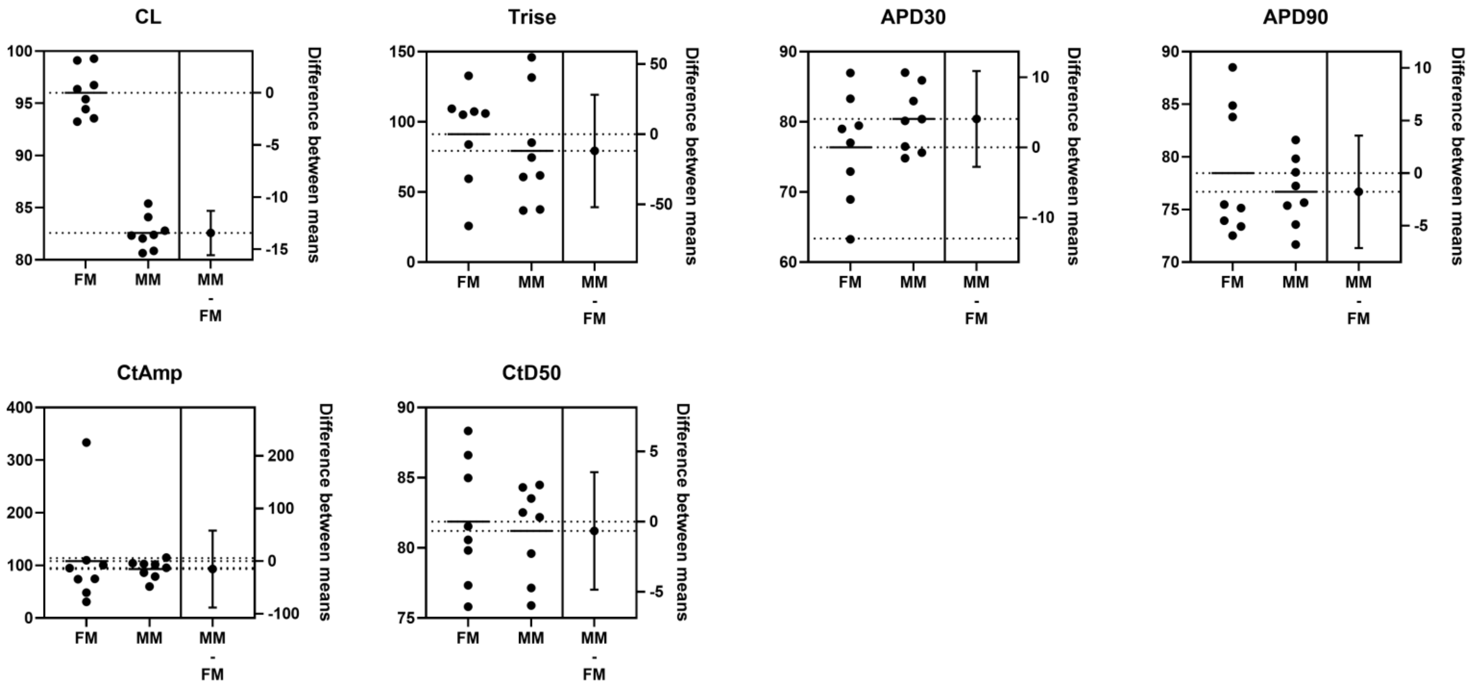

(c) 10µM Milrinone

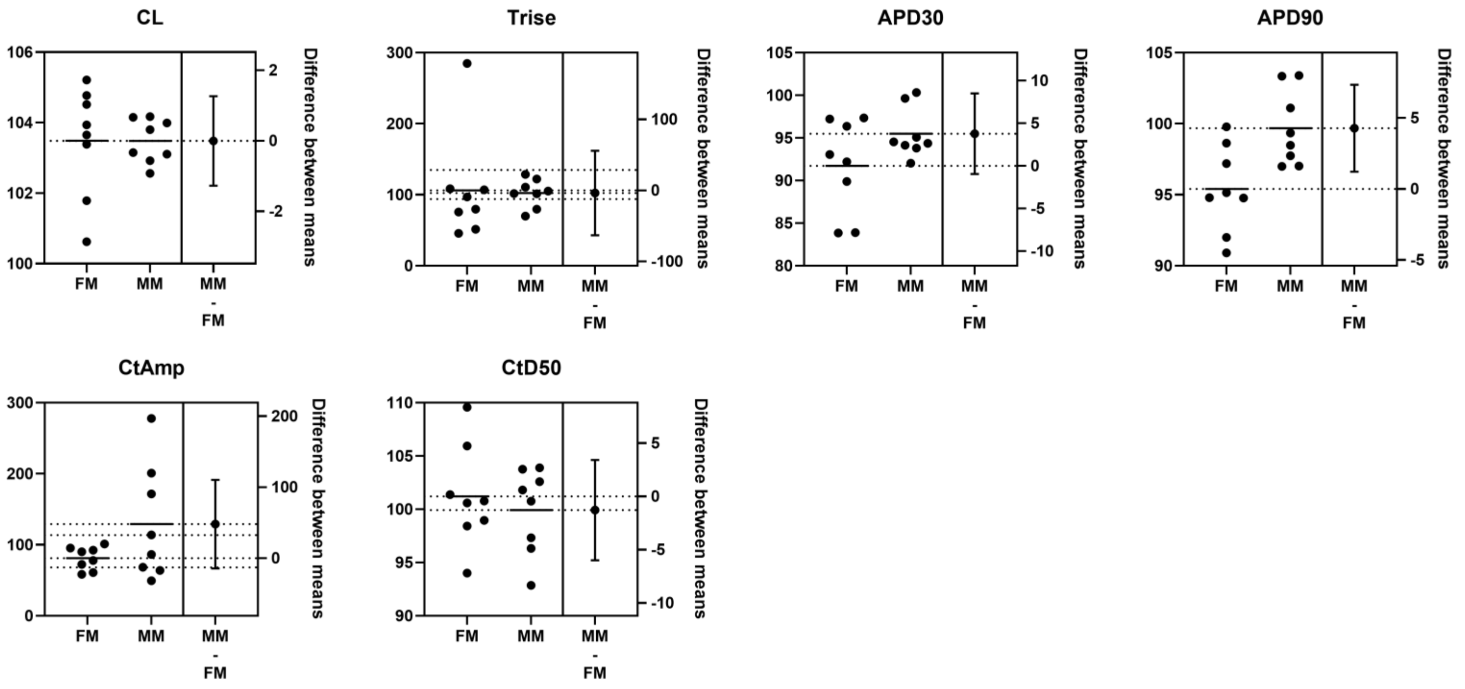

Suppl. Figure S2:

(d) 3μM Levosimendan

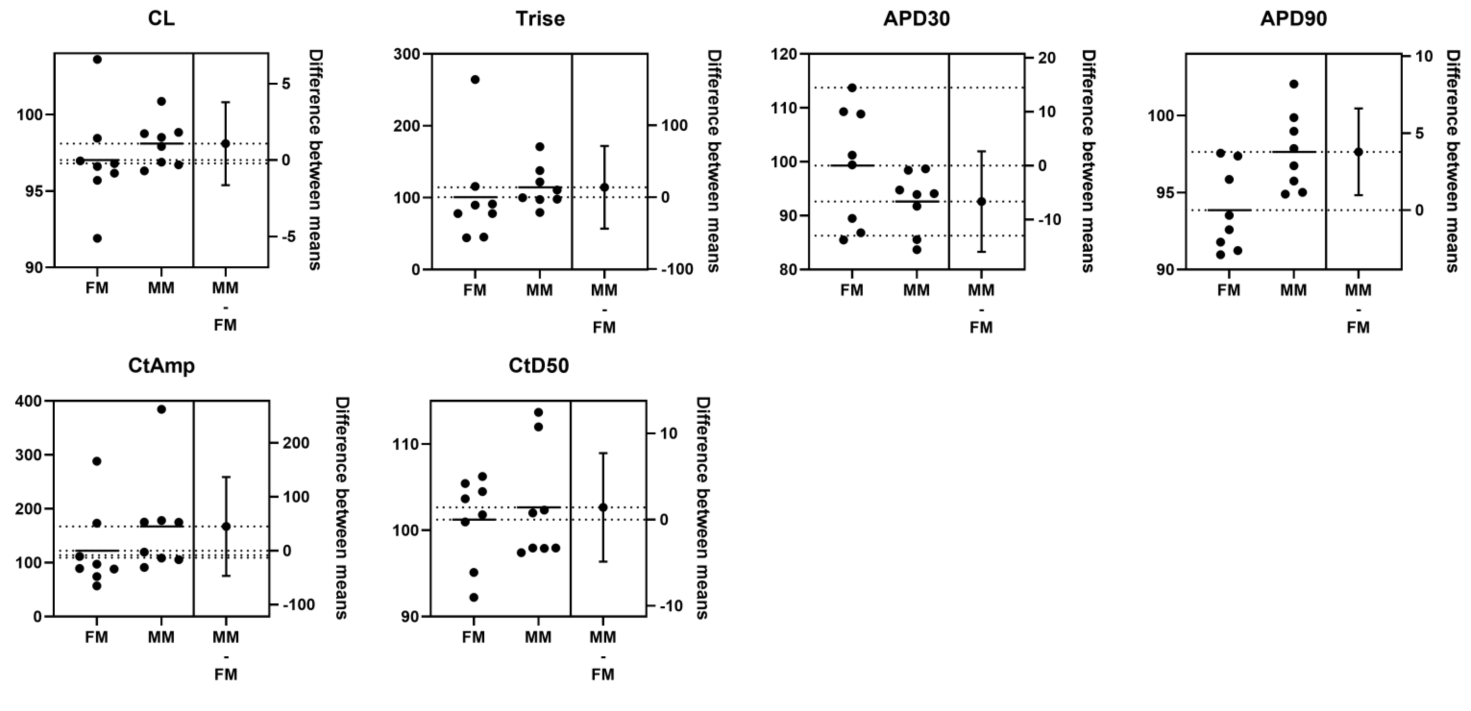

(e) 0.1μM Digoxin

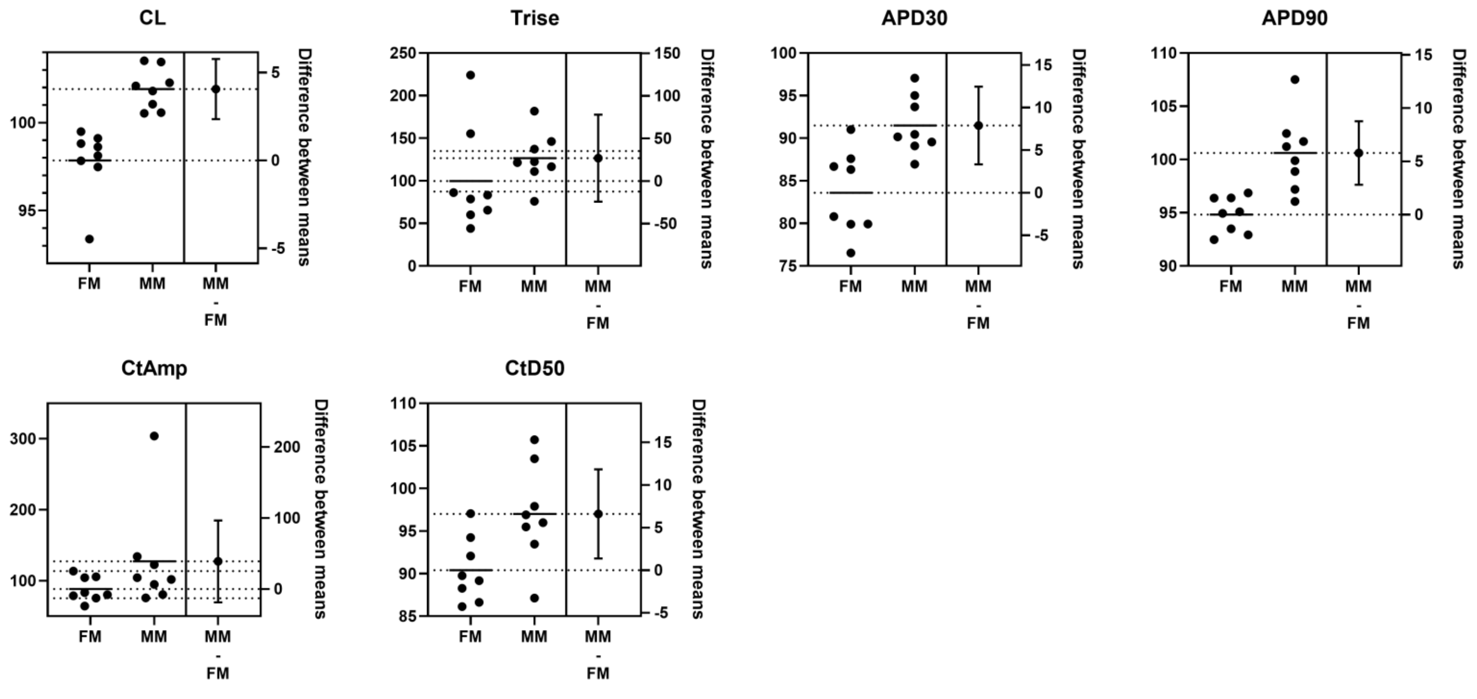

(f) 1μM Carbachol

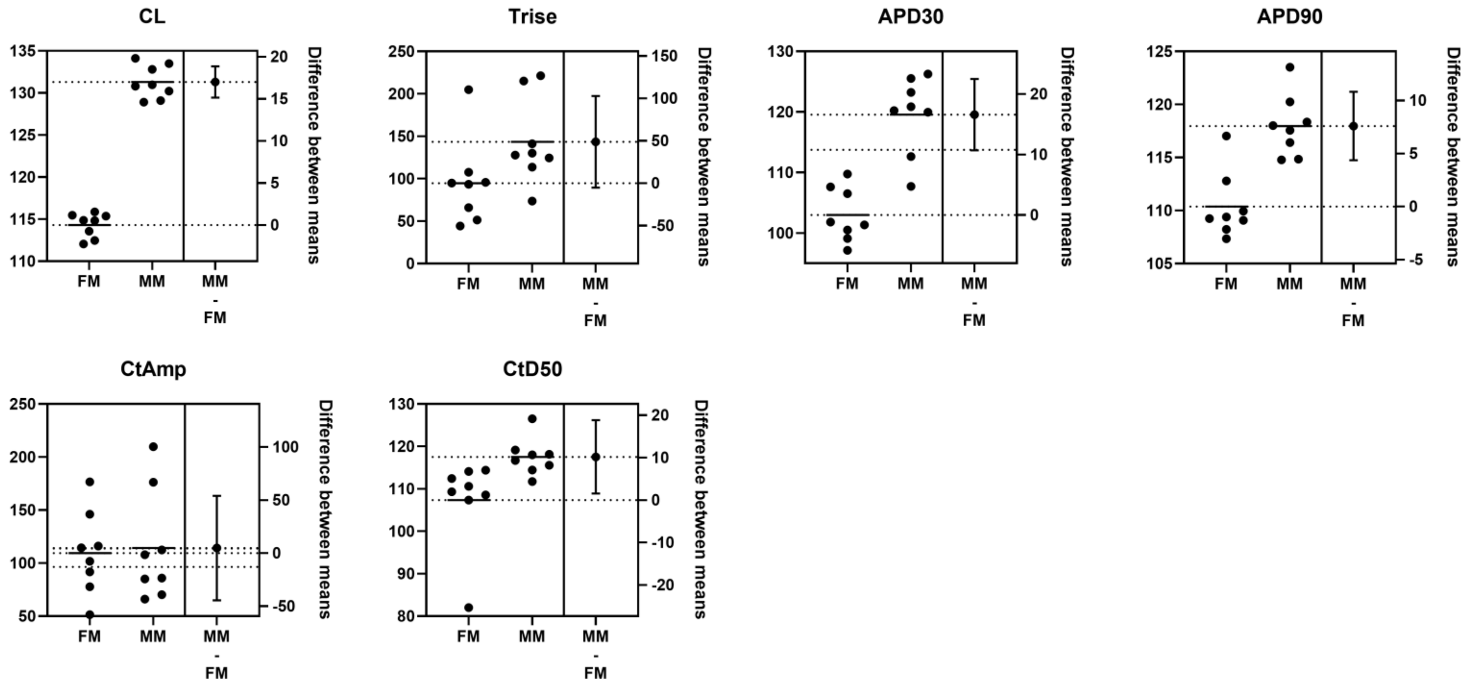

Suppl. Figure S2:

(g) 3μM Ceterizine

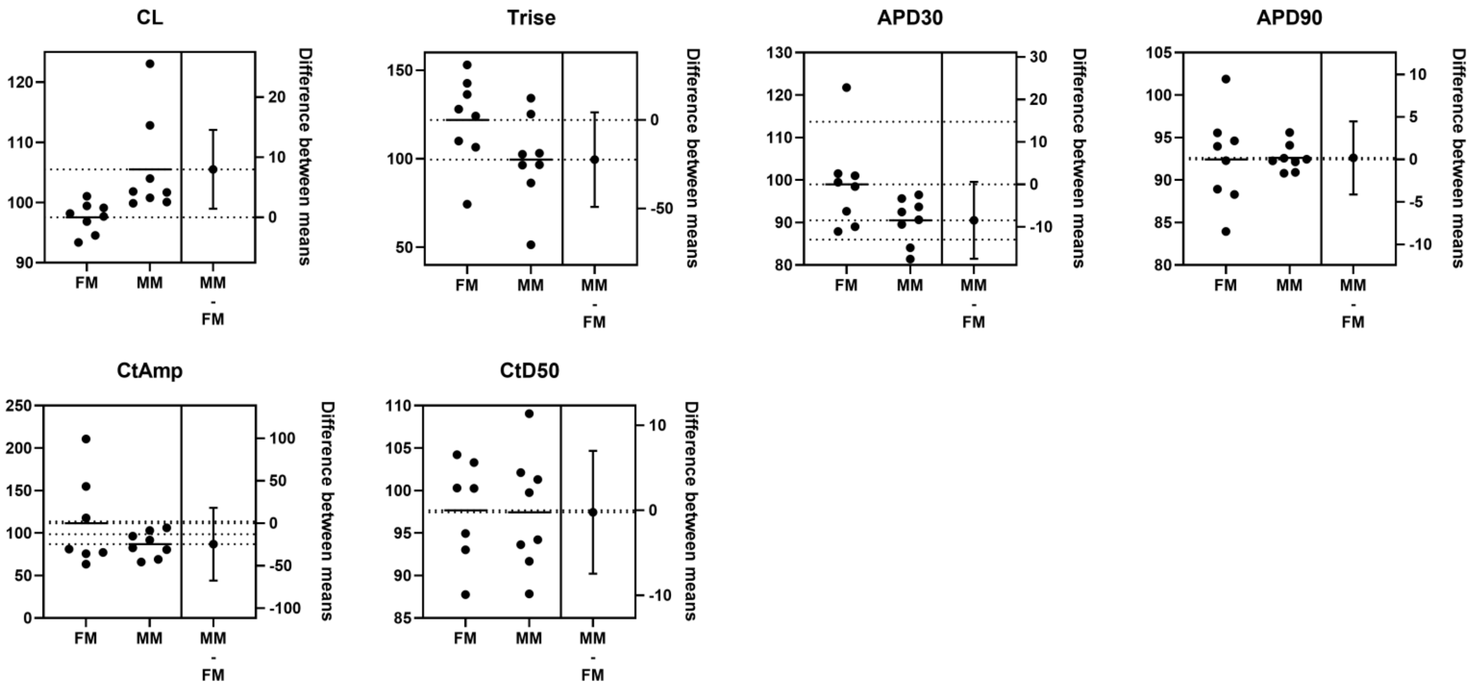

(h) 3μM Oxotremorine

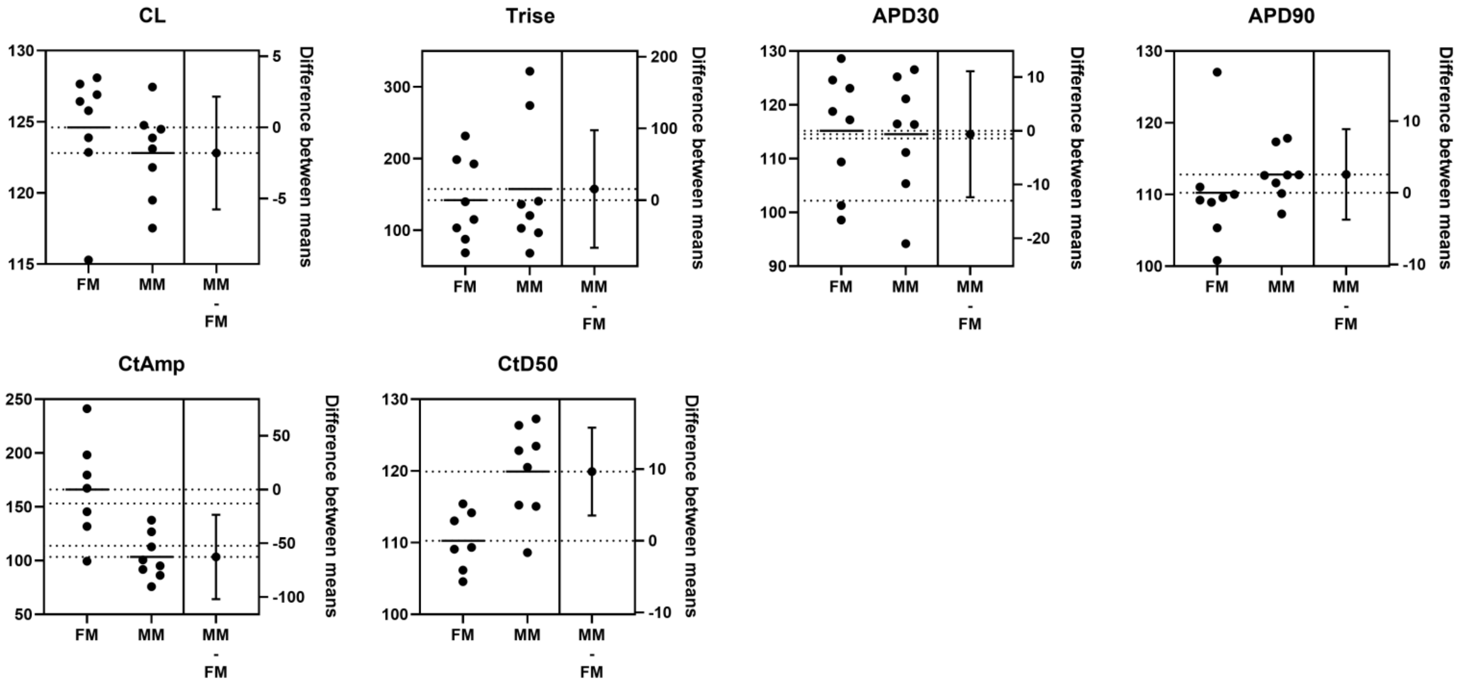

(i) 10μM Dobutamine

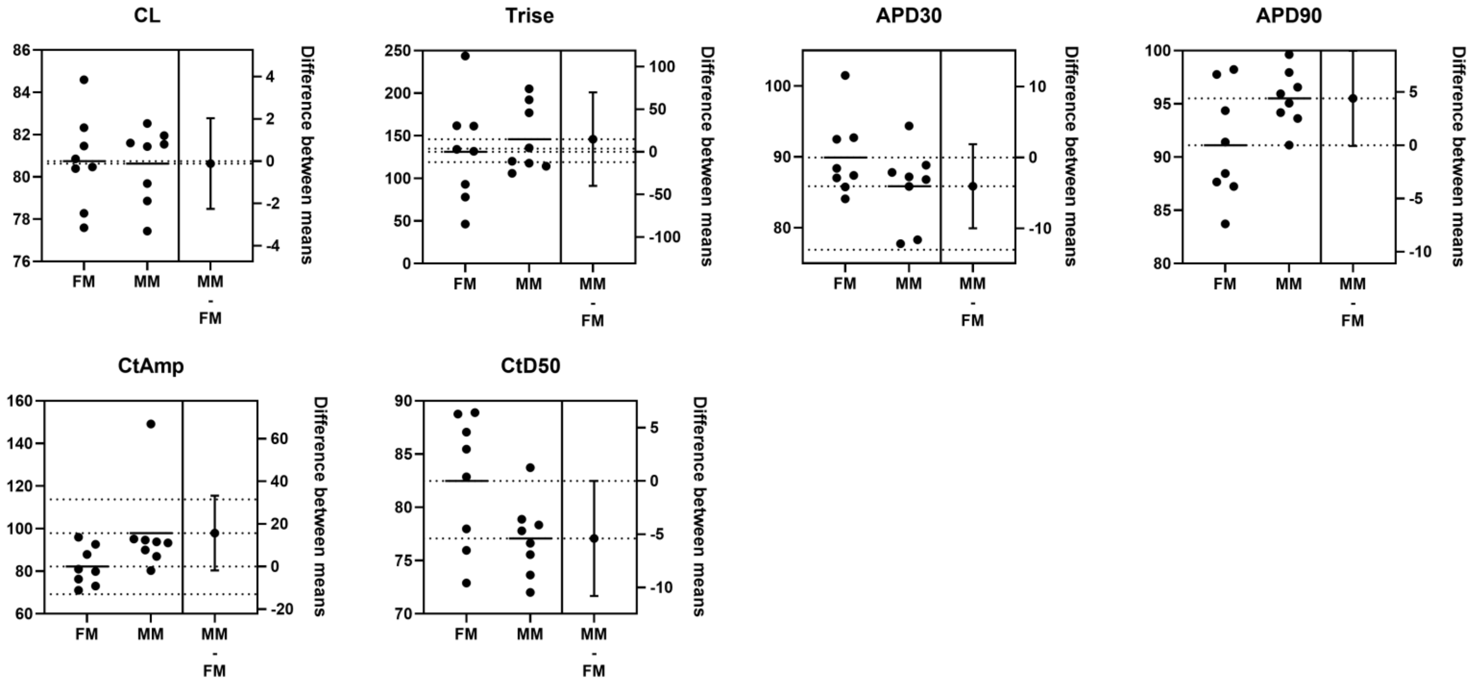

Suppl. Figure S2:

(j) 1µM PA-6

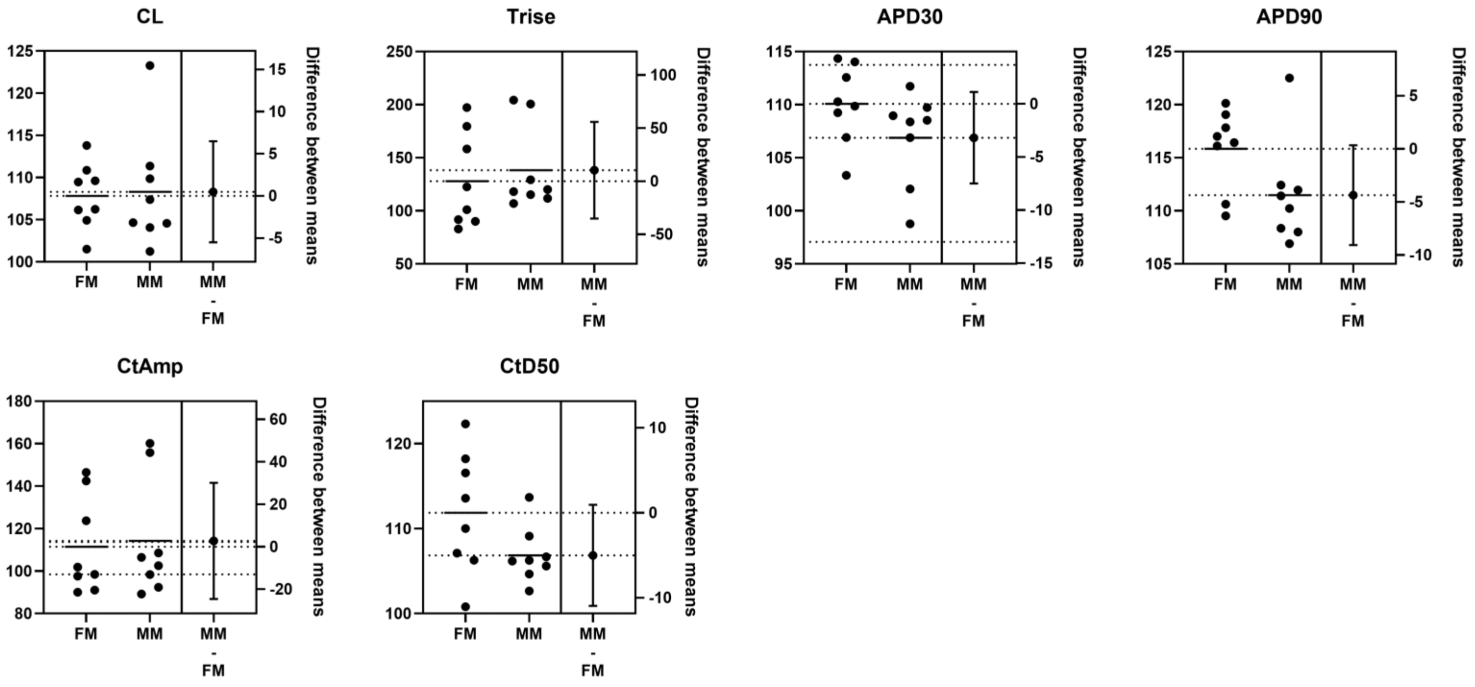

(k) 0.1µM Isoprenaline

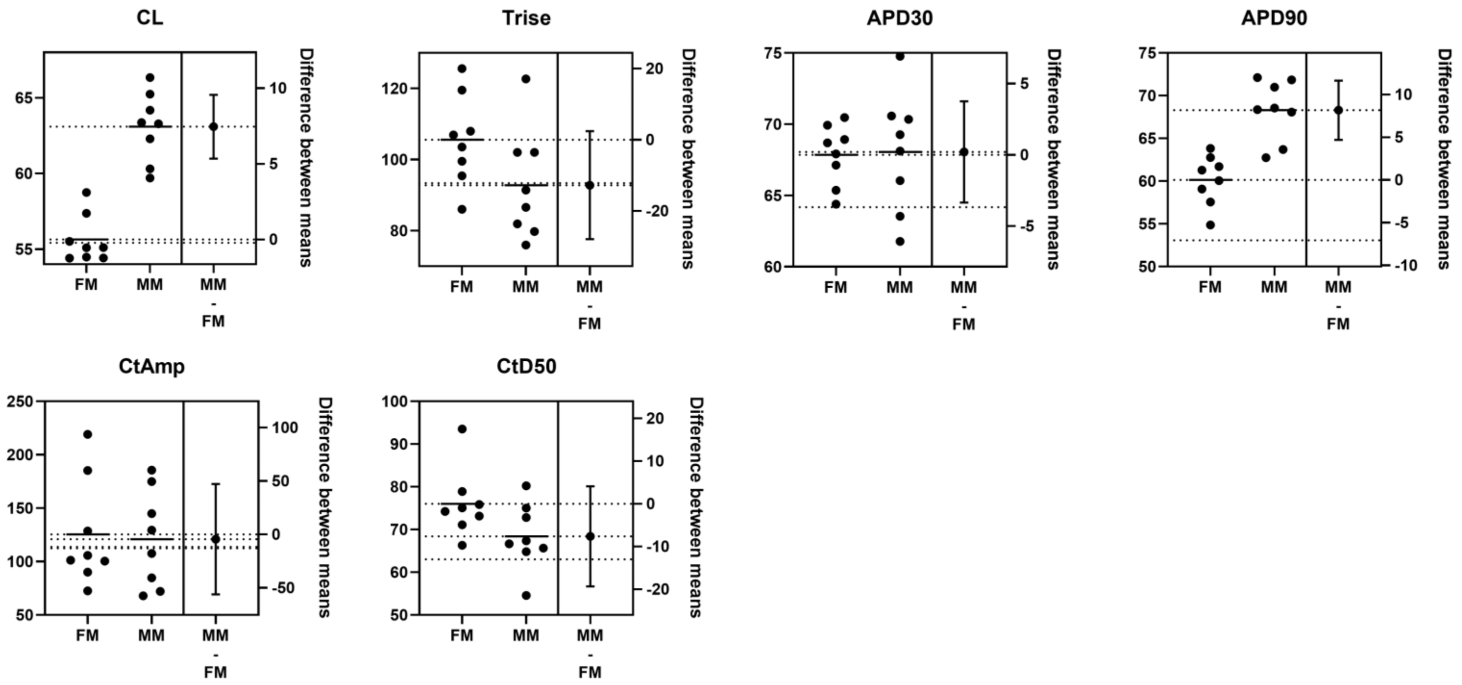

Supplement: Supplementary file 1 [file biomolecules-13-00676-s001.zip › Suppl Fig S2.pdf]
