# Supplementary figures and images for: Does Enhanced Structural Maturity of hiPSC-Cardiomyocytes Better for the Detection of Drug-Induced Cardiotoxicity?
Source: Biomolecules. 2023 Apr 14;13(4):676. doi: 10.3390/biom13040676 (PMC10135569; doi:10.3390/biom13040676)

Suppl. Figure S1:

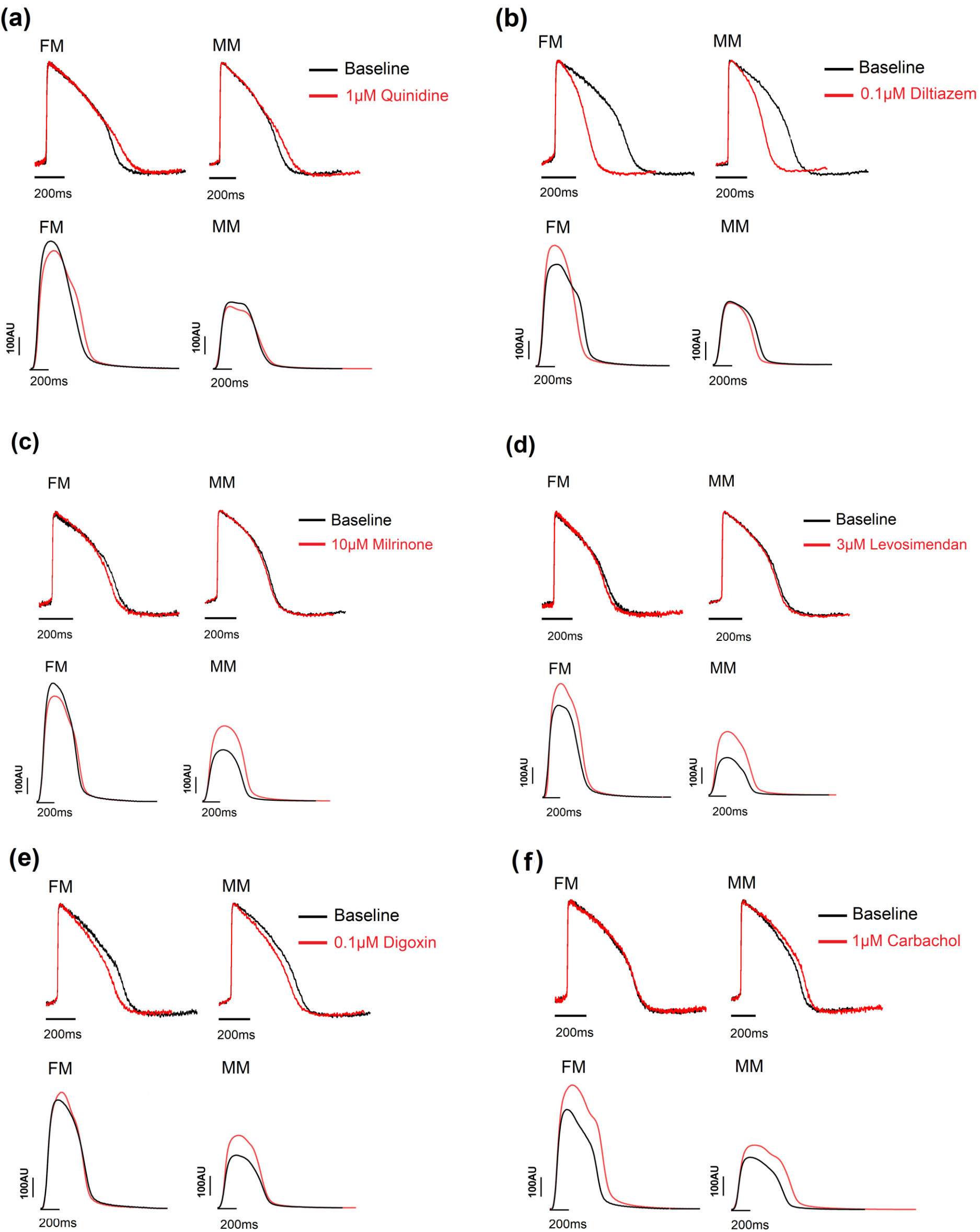

Suppl. Figure S1:

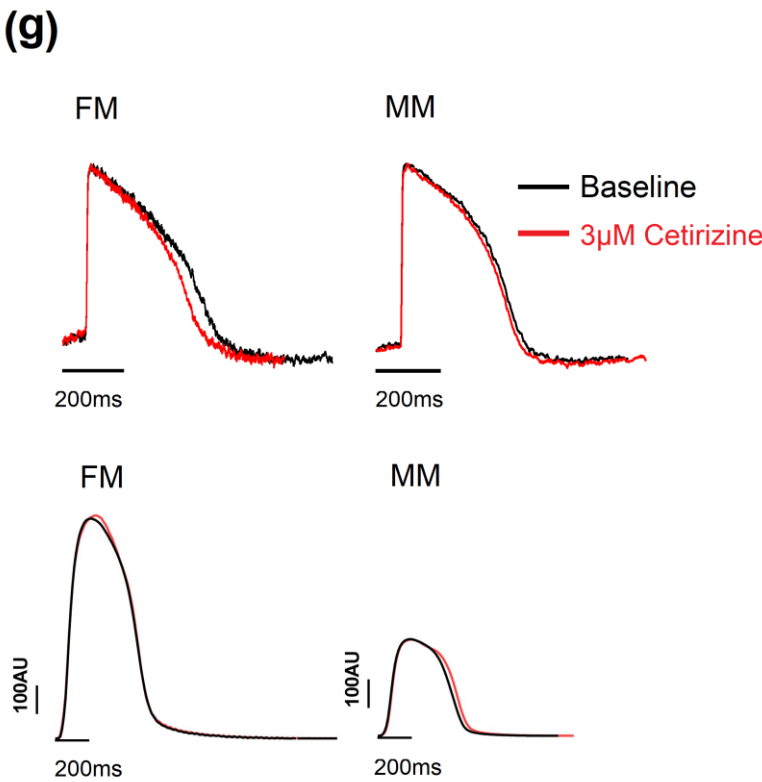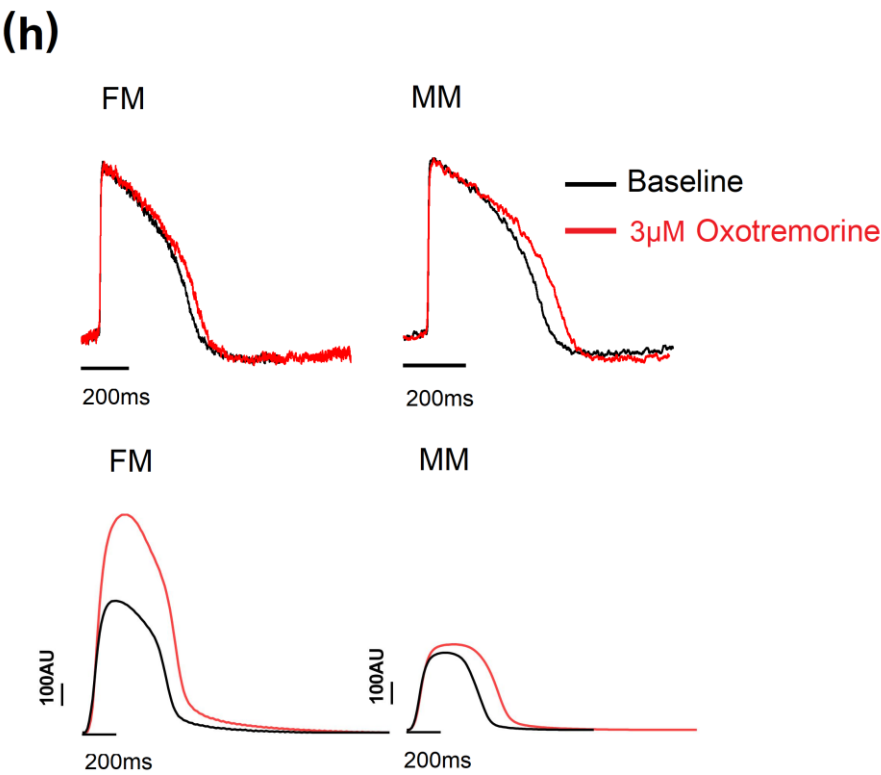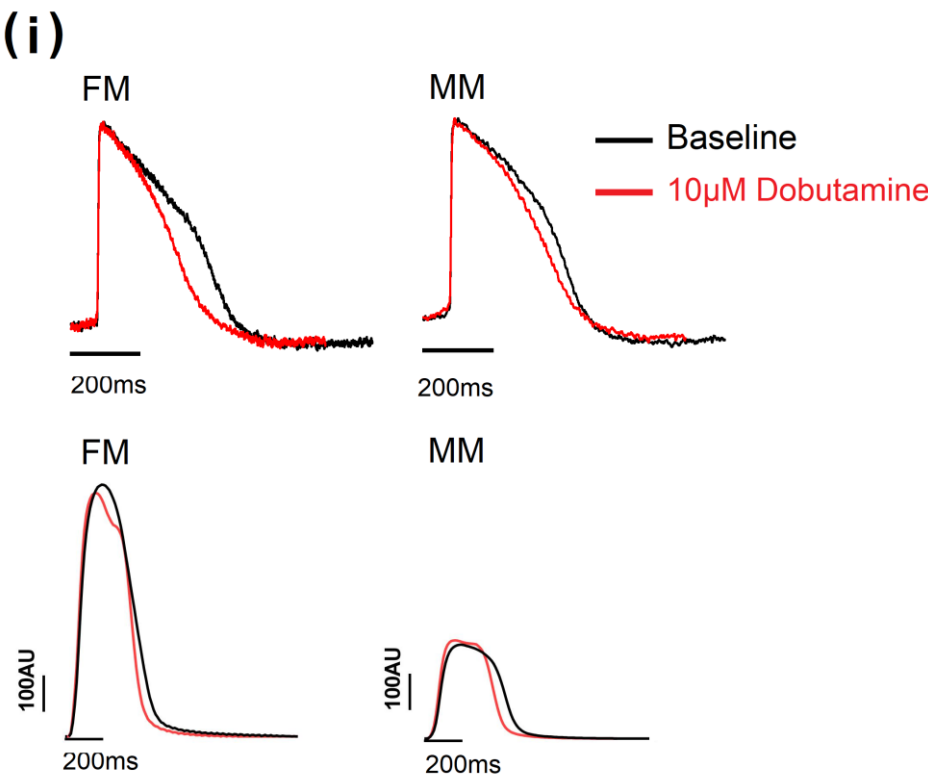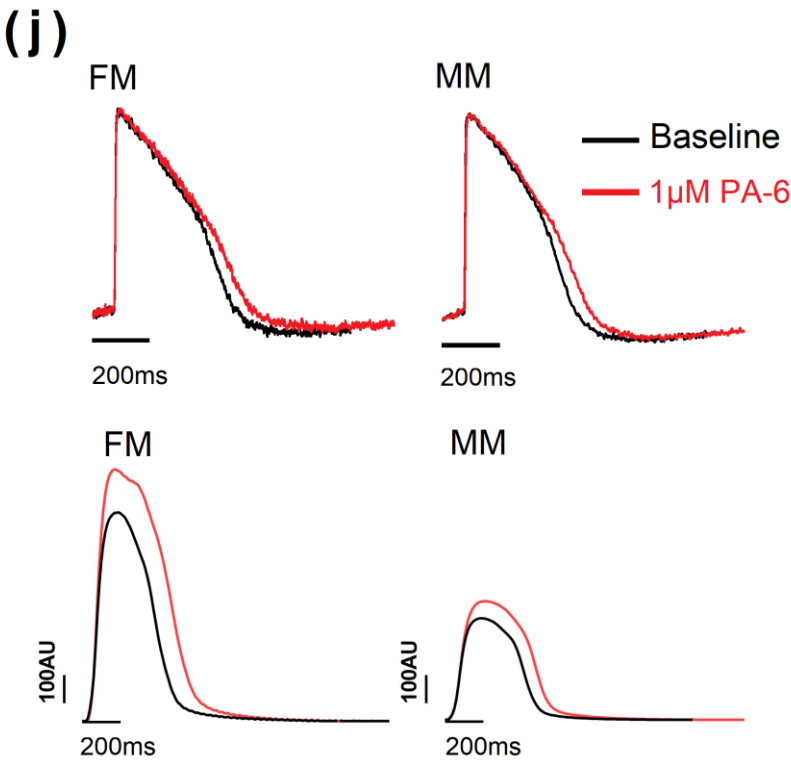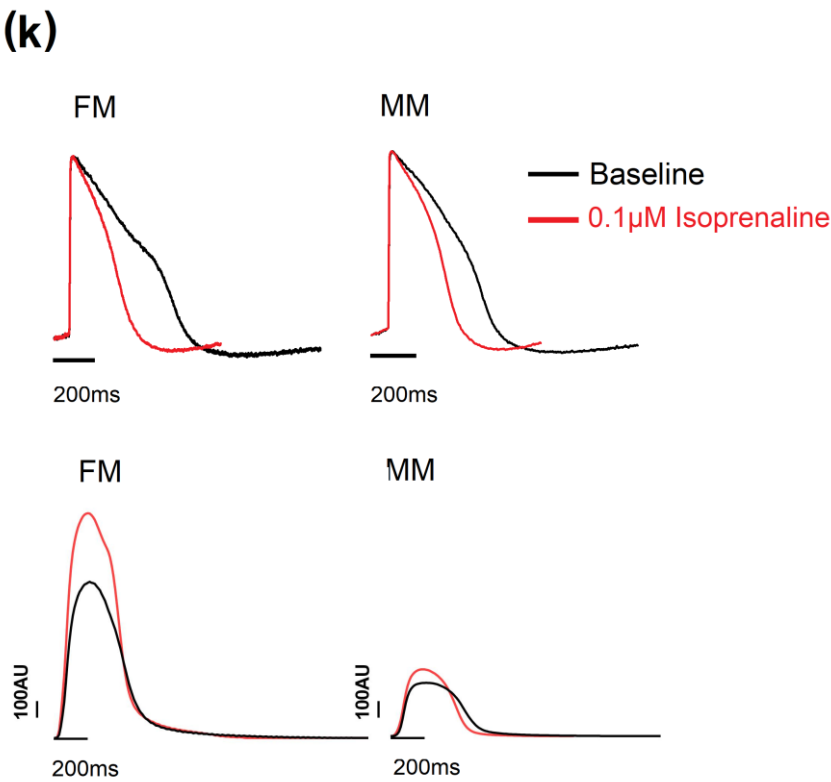

Supplement: Supplementary file 1 [file biomolecules-13-00676-s001.zip › Suppl Fig S1.pdf]
